# Microplastic content of over-the-counter toothpastes - a systematic review

**DOI:** 10.12688/f1000research.132035.1

**Published:** 2023-04-13

**Authors:** Kavery Chengappa S, Ashwini Rao, Aparna K S, Praveen S Jodalli, Ramya Shenoy Kudpi

**Affiliations:** 1 Public Health Dentistry, Manipal College of Dental Sciences, Mangalore, Manipal Academy of Higher Education (MAHE), Manipal, India

**Keywords:** in vitro study, microbeads, microplastics, systematic review, toothpaste

## Abstract

**Background: **Microplastic particles are used as ingredients in personal care products such as face washes, shower gels and toothpastes and form one of the main sources of microplastic pollution, especially in the marine environment. In addition to being a potential pollutant to the environment, the transfer of microplastics to humans can become a severe threat to public health. This systematic review was conceptualized to identify evidence for the presence of and characteristics of microplastics in toothpaste formulations.

**Methods: **The PICOS Criteria was used for including studies for the review. Electronic databases of Scopus, Embase, Springer Link, PubMed, Web of Science and Google Scholar were searched, as well as hand and reference searching of the articles was carried out. The articles were screened using the software application, Covidence® and data was extracted.

**Results: **This systematic review showed that toothpastes from China, Vietnam, Myanmar and the UAE, reported no evidence of microplastics and those from Malaysia, Turkey and India reported the presence of microplastics. The shape of the microplastics present in these toothpastes were found to be granular, irregular with opaque appearance and also in the form of fragments and fibers and the percentage weight in grams ranged from 0.2 to 7.24%.

Malaysia releases 0.199 trillion microbeads annually from personal care products into the environment and toothpastes in Turkey release an average of 871 million grams of microplastics annually. Similarly, in India, it has been reported that 1.4 billion grams of microplastic particles are emitted annually from toothpaste.

**Conclusions: **The findings of this systematic review provide evidence that toothpastes, at least in some parts of the world, do contain microplastics and that there is a great risk of increase in the addition of microplastics to the environment by the use of toothpaste.

## Introduction

A future without plastics is becoming more and more difficult for the younger generation to imagine. Because of their durability and lack of biodegradability, plastic materials are both more appealing to humans and harmful to the environment. Environmental experts today are extremely concerned about microplastics. Microplastics are defined by the United Nations Environment Programme as, solid phase materials, less than 5mm in size, water insoluble, nondegradable and made of plastic. ^1^ They are divided into primary and secondary microplastics based on the manner of development. While secondary microplastics are produced when large plastics are broken up into tiny detritus, primary microplastics are plastics designed to have a microscopic size. ^2^ There are various types of primary microplastics such as polypropylene, polymethyl methacrylate, polyethylene terephthalate, polymethyl methacrylate, nylon, and polyethylene. ^3^

Microplastic particles that are manufactured for their use as ingredients in personal care products are called microbeads or cosmetic microplastics and although they are characterized by a size less than 0.8 mm, most of the microbeads are less than 0.1 mm. ^4^ As a result, since traditional wastewater treatment systems are not built to remove microplastics, they bypass these treatment facilities and are subsequently transferred into river and sea waters, where they persist forever because of their non-biodegradable nature. ^5^ Presence of microplastics in sea water, freshwater, fruits and bottled water have been found across countries. ^6^^–^^9^

Where do these microplastics in the environment come from? It was in the 1990’s that it was first recognized that personal care products such as face washes/scrubs, shower gels and toothpastes formed one of the main sources of microplastic pollution, especially in the marine environment. ^10^ These microplastics when subjected to ultraviolet radiation, gets degraded and absorb persistent organic pollutants (POPs) like polychlorinated biphenyls (PCBs), becoming more toxic in the long-term. ^11^ Once these microplastics make their way into the marine environment, they are ingested by marine organisms at the base of the food chain. ^2^ Studies have reported toxic effects, such as structural changes to the gills, necrosis in other tissues as well as a heightened immune response in mussels that were exposed to microplastics in water bodies. ^12^^,^^13^

In addition to being a potential pollutant to the environment, ^3^^,^^14^ the transfer of microplastics to humans via their engulfment by aquatic animals can become a severe threat to public health. ^15^ Studies have not only identified microplastics in human placenta in utero ^16^ but have also shown evidence of interactions between microplastics and gut epithelium leading to oxidative impairments and disturbance in the inflammatory intestinal balance, impacting the epithelial permeability of the gut and toxicity of immune cells. ^17^ It has been reported ^18^ that prolonged use of toothpaste containing microbeads aggravates the inflammatory process of the gingiva and that the risk of these being unintentionally ingested on a day-to- day basis could also be potentially dangerous to health. ^3^

Although many studies have confirmed the presence of microbeads in face washes/scrubs in significant quantities, ^11^^,^^19^^–^^21^ very few evidence exists ^22^^–^^24^ with respect to the presence of microbeads and their characteristics in toothpaste.

Lebreton and Andrady ^25^ have reported that 60–99 million metric tonnes of mismanaged plastic waste were produced worldwide in 2015 and have predicted that the world’s microplastic emission would triple by 2060 to reach up to 170–270 million metric tons/year, thus portraying a bleak picture. Considering these glaring evidence, the United Nations Environment Program has recommended an eventual phase-out and ban of microplastics in personal care products and cosmetics. ^1^

However, is it even achievable? Microplastics were originally used in personal care, cosmetics, and cleaning products (PCCPs) as abrasives for their scrubbing effects. But now they are believed to carry out several other crucial functions, including acting as binders, bulking agents, emulsifiers, exfoliants, film formers, viscosity regulators, opacifying agents, glitters, skin conditioners, tooth polishing agents, moisturisers, and stabilizers. ^26^

This systematic review was conceptualized to identify evidence for the presence of microplastics in toothpaste formulations, with the hope that this evidence might help in understanding where we stand in our goal of phasing out and eliminating microplastics from toothpaste formulations all over the world. The findings would have implications for research as well as policy implications too.

### Rationale

There is evidence to show that microplastics are present in personal care products like face washes and toothpastes. ^11^^,^^19^^–^^24^ The constituents in these products constitute micropollutants, due to their capacity to cause adverse effects on health and on the environment. These products, especially toothpastes, could be potentially dangerous ^3^ since they could not only be unintentionally ingested on a day-to-day basis but are also a source of environmental pollution as they are carried in the water.

### Objective

To identify relevant literature that assesses the presence and characteristics of microplastics in toothpastes and to analyse and integrate the evidence in a systematic manner.

### Focus questions

This review tries to answer the following questions:

• Do toothpastes contain microplastics?
• If present, what are the characteristics of these microplastics?

## Methods

### Eligibility criteria

**Inclusion criteria**

The PICOS Criteria was used for including studies for the review:

i. Population/participants and conditions of interest: Toothpaste samples
ii. Interventions/exposures: None
iii. Comparisons or control groups: Any or no comparison
iv. Outcomes of interest: Presence and characteristics of microplastics
v. Study designs: *In-vitro* studies

**Exclusion criteria**

The review excluded studies that were published in languages other than English and those studies whose abstracts or full text were unavailable.

### Information sources

The electronic databases of Scopus, Embase, Springer Link, PubMed, Web of Science and Google Scholar were searched. Hand and reference searching of the articles was also carried out.

### Search strategy

The search strategy involved use of the following key terms including the Boolean ‘OR’ and ‘AND’ operators: “microbeads” OR “microplastics” AND “toothpastes” sort by: relevance, Filters: English and the search was carried out between June and December 2022.

### Selection process

The articles were selected and compiled by two reviewers (KC and AR), from these databases and assessed based on the inclusion criteria. The software application, Covidence ^®^ (Covidence, RRID:SCR_016484) URL: https://www.covidence.org/home) was utilised for the selection process.

### Data collection process

Two reviewers, reviewer number 1 (KC) and reviewer number 2(AR), independently screened the articles. Disagreements were resolved with the help of a third reviewer (AKS). The software application, Covidence ^®^ (Covidence, RRID: SCR_016484) URL: https://www.covidence.org/home) was utilised for the process of screening and extraction of data.

### Data items

Data was sought for the outcomes namely, content and characteristics of microplastics in toothpastes. The data extraction template by Covidence was customized for this review and the review was reported according to the standards of Preferred Reporting Items for Systematic reviews and Meta-Analyses (PRISMA) 2020. ^27^

### Quality assessment

Using the ‘Quality Assessment Tool for *In vitro* Studies’ (QUIN), ^28^ the chosen papers’ quality was evaluated. This tool has 12 criteria that must be assessed and gives a point value: adequately specified = 2, inadequately specified = 1, and not specified = 0. The criteria that did not apply were excluded from the calculation of scores. The first reviewer (KC) and second reviewer (AR) each independently evaluated the papers’ quality. Discussions were held to reach an agreement if there were any disagreements.

Using the algorithm, “Final score = (Total score×100)/(2×number of criteria applicable)”, the scores were then combined to get a final score based on which the studies were graded. A score of >70% indicated a low risk of bias in the article, a score of 50% to 70% indicated a medium risk of bias, and a score of <50% indicated a high risk of bias.

## Results

### Study selection

A total of 905 articles were obtained, of which, 695 were from Google Scholar, 128 from Springer Link, 23 from Embase, 22 from PubMed, 20 from Scopus and 17 from Web of Science.

**Figure 1.**
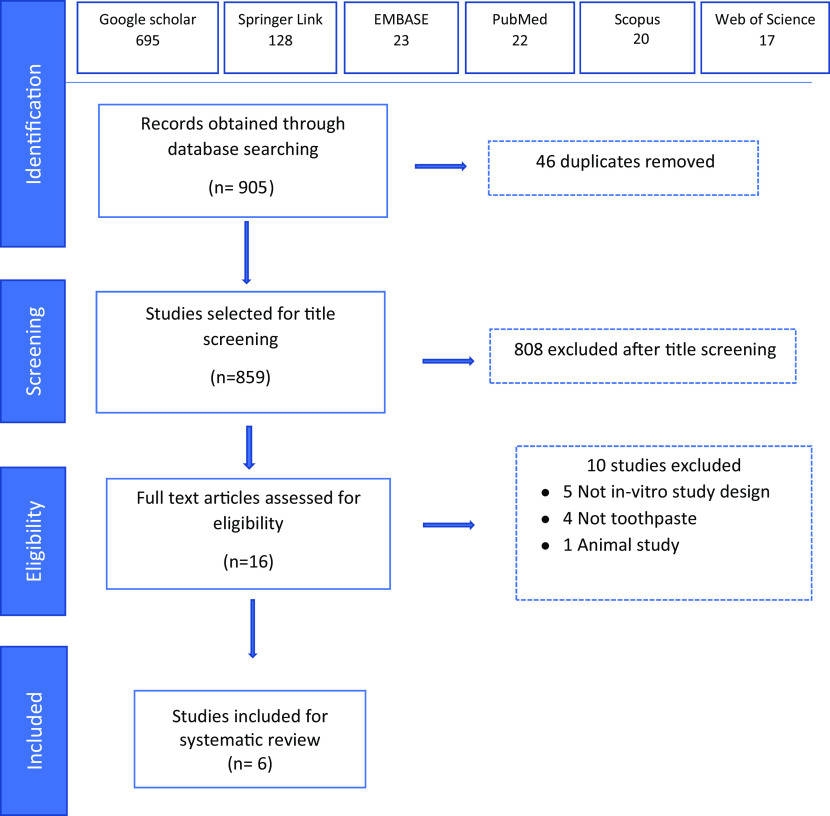
Flow chart of steps in literature search.

Identification of duplicates was done, resulting in the removal of 46 articles and 859 articles were selected for level 1 title screening. After title screening, 808 articles were excluded since they did not confirm with our inclusion criteria and 16 articles were found to be eligible for the next process of full text review. When the full text review was carried out, ten articles were excluded and finally six studies were included for systematic review ( Figure 1).

### Characteristics of included studies

The studies included and their characteristics are summarized in Table 1. Although no filter of ‘Results by year’ was applied, keyword search showed that evidence was available only since 2017.

**Table 1.** Characteristics of included studies.

	Author, Year	Samples with microplastics	Location	Methodology	Composition	Particle size	Shape	Weight in the product in %	Environmental risk assessment
**1**.	Lei *et al.*, 2017	0/135 toothpastes from 23 brands	China	Fourier transform infrared spectrometry (FT-IR) and micro-Raman spectroscopy (Raman)	None of the toothpastes in their samples contained MPs	-	-	-	-
**2**.	Praveena *et al.*, 2018	1/5	Malaysia	FTIR spectroscopy, dynamic light scattering measurement in microscope	Low Density Polyethylene	3–145 μm	Granular	7.24%	five facial cleansers and five toothpastes released 0.199 trillion microbeads per year
**3**.	Ustabasi and Baysal 2019	4/20	Turkey	FTIR and microscopic analysis	Polyethylene	4–20 μm	irregular shapes with opaque appearance	0.4-1%	Yearly 871 million g of MPs on average is estimated to be emitted from toothpastes in Istanbul, Turkey
**4**.	Mon and Nakata 2020	0/3	China, Vietnam and Myanmar	FTIR (Fourier transform infrared spectrophotometer) and ATR (Attenuated total reflection) was used	None of them had any microplastics	-	-	-	-
**5**.	Elkashlan *et al.*, 2022	0/33	31 of the toothpastes samples were from UAE markets and 2 were imported from Syria	FT-IR and EDS (energy dispersive X-ray spectroscopic) analysis	None of them had any microplastic particles	-	-	-	-
**6**.	Madhumitha *et al.*, 2022	10/10	India	Extracted by vacuum filtration and characterized with and FTIR (Fourier transform infrared spectrophotometer) and microscopic analyses	Four major polymer types, viz., cellophane, polypropylene, polyvinyl chloride, and polyamide	3.5 μm with a maximum size exceeding 400 μm size range of <100 μm, 100–400 μm, and >400 μm	Colorless fragments and fibers.	0.2 to 0.9%	Average MPs emission for India was calculated as 1.4 billion g/year

The number of toothpastes samples included, varied from a minimum of three in the study by Mon and Nakata ^29^ to a maximum of 135 samples of toothpastes belonging to 23 brands, in the study by Lei *et al.* ^3^ These studies reported the type of microplastic present along with the particle size, shape, color and particle weight. While most studies used Fourier Transform Infrared Spectrometer (FTIR), some studies further used ATR (Attenuated total reflection) and microscopic analysis ^11^ to identify the type and characteristics of the microplastic particles in toothpastes.

Out of the 6 studies selected for the final review, three studies, with toothpastes from China, ^3^^,^^29^ Vietnam, ^29^ Myanmar ^29^ and the UAE, ^30^ reported no evidence of microplastics in the toothpaste samples that were analyzed. Among the remaining three studies, two studies, one from Malaysia ^22^ and another from Turkey ^23^ reported the presence of microplastics with composition of polyethylene whereas the study from India ^24^ reported the presence of microplastics with composition of cellophane, polypropylene, polyvinyl chloride and polyamide.

Although microplastics are defined as water insoluble, nondegradable plastic less than 5mm in size, the particle sizes of microplastics were found to range from 3.5 μm in the study by Madhumitha *et al.* ^24^ from India to 4–20 μm in the study by Ustabasi and Baysal ^23^ in Turkey and 3–145 μm in the study by Praveena *et al.* ^22^ in Malaysia.

Regarding the shape of the microplastics present in toothpastes, it was reported to be granular, ^22^ irregular with opaque appearance ^23^ and as colorless fragments and fibers. ^24^

The weight of microplastics in the product was reported as 7.24 % by Praveena *et al.*, ^22^ whereas Ustabasi and Baysal ^23^ and Madhumitha *et al.* ^24^ reported a range of 0.4–1% and 0.2–0.9% respectively.

Three studies ^22^^–^^24^ also did the environmental risk assessment for microplastics released through the toothpaste. Praveena *et al.* ^22^ reported that five facial cleansers and five toothpastes released 0.199 trillion microbeads per year in Malaysia. Ustabasi and Baysal ^23^ reported an estimated average of 871 million grams of microplastics released every year from toothpastes in Turkey. Madhumitha *et al.* ^24^ have reported an average yearly emission rate of 1.4 billion grams of microplastic particles from toothpaste in India.

### Quality assessment

Using the ‘Quality Assessment Tool for *In vitro* Studies’ (QUIN), the selected articles were assessed and graded to obtain the risk of bias. Out of the 12 criteria given by QUIN, three were excluded since these criteria did not apply to this review and so the studies were graded based on 9 criteria. All the studies showed a medium risk of bias since the final scores ranged from 50–70%. Most of the articles had adequately specified the aims/objectives, given detailed explanation of methodology, method of measurement of outcome, statistical analysis and presented the results adequately. The most common criteria which were not specified included, detailed explanation of sampling size calculation and the sampling technique. The criteria of outcome assessor details and blinding was also not specified in all the six selected articles which led to all studies being categorized as showing medium risk of bias ( Table 2).

**Table 2.** Quality assessment of the selected articles using the Quality Assessment Tool for *In vitro* Studies (QUIN).

	Criteria	Lei *e* *t al.*, 2017 ^3^	Praveena *et al.*, 2018 ^22^	Ustabasi and Baysal 2019 ^23^	Mon and Nakata 2020 ^29^	Elkashlan *et al.*, 2022 ^30^	Madhumitha *et al.*, 2022 ^24^
1.	Clearly stated aims/objectives	2	2	2	1	1	2
2.	Detailed explanation of sample size calculation	0	1	0	0	0	0
3.	Detailed explanation of sampling technique	0	1	0	0	0	0
4.	Details of comparison group	Excluded					
5.	Detailed explanation of methodology	2	2	2	2	2	2
6.	Operator details	Excluded					
7.	Randomization	Excluded					
8.	Method of measurement of outcome	2	2	2	2	2	2
9.	Outcome assessor details	0	0	0	0	0	0
10.	Blinding	0	0	0	0	0	0
11.	Statistical analysis	2	2	2	2	2	2
12.	Presentation of results	2	2	2	2	2	2
	SUM	10	12	10	9	9	10
	FINAL SCORE	55.6	66.7	55.6	50	50	55.6
	RISK OF BIAS	Medium	Medium	Medium	Medium	Medium	Medium

## Discussion

Microplastics in water are susceptible to physical forces, fluctuating temperatures, ultraviolet radiation, oxidation, and salinity. They also become coated with bacterial biofilm. Since they settle to the bottom and have altered surface morphologies and higher densities, these weathered or conditioned microplastics are more readily available to a wide range of marine organisms. ^31^ However, polyethylene is a type of microplastic that floats on the water’s surface due to its specific density of <1, ^32^ and therefore it is available to a range of planktonic species as well as fish and seabirds that eat at the water’s surface.

Although evidence shows that microplastics are contaminating our environment in great proportion and constitute an environmental hazard leading to health impacts, they are still being incorporated in personal care products including toothpastes. Our focus was on microplastics in toothpastes, and we found a relative dearth of studies in this area. Only six studies fulfilled our inclusion criteria and were included in this systematic review, showing evidence only from a few countries like China, ^3^^,^^29^ Malaysia, ^22^ Turkey, ^23^ Vietnam, ^29^ Myanmar, ^29^ UAE ^30^ and India, ^24^ out of which 3 studies ^22^^–^^24^ showed evidence of their presence in toothpaste.

The existence of microplastics was reported in three investigations, two ^22^^,^^23^ of which identified polyethylene as the microplastic while the third, an Indian study, ^24^ identified cellophane, polypropylene, polyvinyl chloride, and polyamide. Interestingly, the samples from India did not include polyethylene, a common polymer found in toothpastes in other studies.

The size of the microplastic particles found in the selected studies ranged from 3.5 μm to 145 μm. Because of their small size, microplastics flow through wastewater systems and are probably not detected by wastewater treatment facilities. Microplastics are available to microscopic species at the base of the food chain because of their limited size range and resistance to degradation in the environment. As a result, they enter the marine environment where they are consumed by marine life and then they enter the human body. ^11^

Regarding the shape of the microplastics present in toothpastes, studies reported them to be granular, ^22^ irregular in appearance ^23^ and as fragments and fibers. ^24^ Evidence ^33^ shows that these microplastics are not always spherical but have a variety of irregular shapes. Despite the fact that the word “bead” refers to spherical particles and despite the widespread misconception that microbeads are the coloured spherical microparticles found in toothpastes, ^11^ microbeads are typically of irregular form to improve abrasion. ^4^^,^^34^ In reality, PCCPs may contain multiple types of bead shapes, and irregular shapes contribute to larger specific surface areas and therefore more friction. ^35^

Regarding the color of the microplastics present in toothpastes, one study ^23^ reported opaque appearance and another ^24^ as colorless. Microbeads have been shown to have different colors such as white, transparent, opaque, blue, red and orange. ^11^

Three studies ^22^^–^^24^ also did the environmental risk assessment for microplastics released through the toothpaste. Praveena *et al.* ^22^ reported that five facial cleansers and five toothpastes released 0.199 trillion microbeads per year in Malaysia. Ustabasi and Baysal ^23^ reported that an estimated average of 871 million grams of microplastics are released every year from toothpastes in Turkey. According to Madhumitha *et al.*, ^24^ in India, 1.4 billion grams of microplastic particles are emitted from toothpaste each year on an average. Using toothpaste might carry a significant danger of increasing the amount of microplastics that are added to the environment. ^36^

The quality assessment of the selected articles showed that all the studies were classified under medium risk of bias. None of the articles gave any detailed explanation of the sample size calculation and the sampling technique. The criteria of outcome assessor details and blinding was also not specified in all the six selected articles. This highlights the need for maintaining scientific rigor in every research irrespective of where they stand in the hierarchy of evidence.

### Limitations of the evidence

There is limited evidence available with respect to the presence of and characteristics of microplastics in toothpastes. Out of the 195 countries in the world, data is available only from a few countries like China, Vietnam, Myanmar, UAE, Malaysia, Turkey and India, and here too, data is not available for all the toothpastes available. More evidence of good quality is needed from all parts of the world to find out the role of toothpastes in adding to the microplastic burden of the environment.

## Conclusions

The result of this systematic review shows that toothpastes, at least in some regions of the world, do contain microplastics and there is a significant danger that using these toothpastes will increase the amount of microplastics added to the environment. Even if it is begun to take steps now, to fully outlaw microbeads internationally, the environment would continue to contain microplastics for a very long time. It is therefore high time to initiate urgent action to curb the menace of microplastics in the environment by eliminating its presence in personal care, cosmetics and cleaning products globally.

### Implications for future research

Evidence from this systematic review shows that very few studies have been done to identify the presence of microplastics in toothpastes. Further research with lower risk of bias needs to be carried out in all parts of the world for conclusively determining the role of microplastics present in toothpaste in contributing to the microplastic burden of the environment, and to determine the possible long-term toxicity of these compounds on the human body.

### Implications for policy

• Source management is a solid strategy to reduce the growing ecological risk posed by microplastics, and since PCCPs are the principal source of primary microplastics, their inclusion should be phased out in favour of more ecologically friendly additives.
• Even though a few nations have passed laws restricting the use of microplastics in healthcare items, many do not enforce these laws strictly. To combat the growing environmental hazard posed by microplastics, a regulatory approach, stricter testing of toothpaste samples before they are let into the market, and stricter enforcement of relevant regulations are urgently required.

### Registration and protocol

Since this was a systematic review of *in vitro* studies, it could not be registered in PROSPERO. However, the review protocol can be found in the Extended data. ^37^

## Data Availability

All data underlying the results are available as part of the article and no additional source data are required. Figshare: Microplastic content of over-the-counter toothpastes – A systematic review, DOI: https://doi.org/10.6084/m9.figshare.22179772.v1. ^38^ The project contains the following extended data:
‐ Protocol.docx Figshare. PRISMA checklist. Microplastic content of over-the-counter toothpastes – A systematic review, DOI: https://doi.org/10.6084/m9.figshare.22179772.v1. ^38^ The project contains the following reporting guidelines:
‐ PRISMA checklist.docx ‐ PRISMA flow chart.docx Data are available under the terms of the Creative Commons Zero “No rights reserved” data waiver (CC by 4.0 Public domain dedication).
