# Supplementary material for: Microplastic content of over-the-counter toothpastes - a systematic review
Source: F1000Res. 2023 Apr 13;12:390. [Version 1] doi: 10.12688/f1000research.132035.1 (PMC10372460; doi:10.12688/f1000research.132035.1)
Supplement: Peer review history

# Peer review: Reviewer response for version 1

**DOI:** 10.5256/f1000research.144931.r189319

**Published:** 2023-07-26

**Authors:** Harsh Priya

**Affiliations:** 1 Division of Public Health Dentistry, Centre for Dental Education and Research, All India Institute of Medical Sciences, New Delhi, Delhi, India

Dear Authors

 Thank you for this manuscript. It is very pertinent for the current times. It is a futuristic clarion call for the much talked about CLIMATE CHANGE with dental fraternity as one of the stakeholders.

 I would suggest following points.

 Abstract:

- Methods section of Abstract - Line "The articles were screened using the software application, Covidence® and data was extracted." can be better framed.
- Results section of Abstract - "0.199 trillion microbeads" - Is the grams / unit missing? Kindly recheck! Else can write ("Microbeads emission in number"/ "Total microbeads emission in number")

Introduction:

- Line: "The findings would have implications for research as well as policy implications too." this sentence can be improved.

Search Strategy:

- Any of the terms used were MeSH terms? Can include MeSH terms!

Regarding the Identification and Visualisation of particles in personal care products the methods have been listed at FTIR and ATR. Further elaboration can be done on this point. For example **size, shape and color **of these polymers were also analyzed using an open-source particle analysis software named ImageJ 1.51 and **composition** of polymers was identified using Thermo Scientific Nicolet 6700 FTIR Spectrometer.

 Thank you

 Best wishes

 Harsh Priya

Are the rationale for, and objectives of, the Systematic Review clearly stated?

Yes

Is the statistical analysis and its interpretation appropriate?

Yes

Are sufficient details of the methods and analysis provided to allow replication by others?

Yes

Are the conclusions drawn adequately supported by the results presented in the review?

Yes

Reviewer Expertise:

Dental Public Health

I confirm that I have read this submission and believe that I have an appropriate level of expertise to confirm that it is of an acceptable scientific standard.

# Peer review: Reviewer response for version 1

**DOI:** 10.5256/f1000research.144931.r169445

**Published:** 2023-05-26

**Authors:** Arun Simon

**Affiliations:** 1 Department of Public Health Dentistry, Sri Ramakrishna Dental College and Hospital, Coimbatore, Tamil Nadu, India

The study looks interesting and pertinent in the current times of heightened environmental pollution concerns. The authors have correctly established the need for this study highlighting the importance of this topic in the current scenario. This review article is a step in the right direction to help build evidence in this topic. The authors have also touched upon on the major databases to select the articles and use of Covidence software gives confidence in their selection process. Overall the conclusions can be believed and looks appropriate for the sated objectives of the study. However the authors can make clear some of the following points

1. During literature search, did they find any article in language other than English?
2. What were the efforts taken to minimize errors in data extraction?

Are the rationale for, and objectives of, the Systematic Review clearly stated?

Yes

Is the statistical analysis and its interpretation appropriate?

Yes

Are sufficient details of the methods and analysis provided to allow replication by others?

Yes

Are the conclusions drawn adequately supported by the results presented in the review?

Yes

Reviewer Expertise:

Dental Public Health and Sociology

I confirm that I have read this submission and believe that I have an appropriate level of expertise to confirm that it is of an acceptable scientific standard.
